# Effects of Acute Blueberry Flavonoids on Mood in Children and Young Adults

**DOI:** 10.3390/nu9020158

**Published:** 2017-02-20

**Authors:** Sundus Khalid, Katie L. Barfoot, Gabrielle May, Daniel J. Lamport, Shirley A. Reynolds, Claire M. Williams

**Affiliations:** School of Psychology and Clinical Language Sciences, University of Reading, Earley Gate, Whiteknights, Reading RG6 7BE, UK; sundus.khalid@pgr.reading.ac.uk (S.K.); k.l.barfoot@pgr.reading.ac.uk (K.L.B.); gabrielle.may@pgr.reading.ac.uk (G.M.); daniel.lamport@reading.ac.uk (D.J.L.); s.a.reynolds@reading.ac.uk (S.A.R.)

**Keywords:** depression, mood, affect, dysphoria, cognition, flavonoid, blueberries, children, young adults

## Abstract

Epidemiological evidence suggests that consumption of flavonoids (usually via fruits and vegetables) is associated with decreased risk of developing depression. One plausible explanation for this association is the well-documented beneficial effects of flavonoids on executive function (EF). Impaired EF is linked to cognitive processes (e.g., rumination) that maintain depression and low mood; therefore, improved EF may reduce depressionogenic cognitive processes and improve mood. Study 1: 21 young adults (18–21 years old) consumed a flavonoid-rich blueberry drink and a matched placebo in a counterbalanced cross-over design. Study 2: 50 children (7–10 years old) were randomly assigned to a flavonoid-rich blueberry drink or a matched placebo. In both studies, participants and researchers were blind to the experimental condition, and mood was assessed using the Positive and Negative Affect Schedule before and 2 h after consumption of the drinks. In both studies, the blueberry intervention increased positive affect (significant drink by session interaction) but had no effect on negative affect. This observed effect of flavonoids on positive affect in two independent samples is of potential practical value in improving public health. If the effect of flavonoids on positive affect is replicated, further investigation will be needed to identify the mechanisms that link flavonoid interventions with improved positive mood.

## 1. Introduction

Major depressive disorder is the leading international cause of disability and is estimated to affect 350 million people worldwide [1]. It is the second most common cause of death in 15–29 years old, via suicide [1]. Current treatments for depression include psychological therapies and a range of pharmacological agents. The treatment options recommended for children and adolescents are limited, with only one recommended pharmacological treatment, fluoxetine, in addition to psychotherapy. These treatment options are further constrained because of concerns about the use of anti-depressant medication with young people, and because most young people do not have easy access to psychological therapies [2,3,4]. Therefore, there is a pressing need for alternative interventions, especially those that offer a cost-effective and practical means of preventing, or alleviating, depression in this population.

A common symptom of depression is impaired cognitive functioning, with significant deficits in executive functioning (EF). EF is an umbrella term, describing cognitive processes such as working memory, planning, problem-solving, cognitive flexibility, inhibitory control, directing attention, thoughts and, therefore, behaviours. Impaired EF is believed to maintain depressive symptoms, such as negative self-perception and low mood, via perseveration and rumination [5,6,7,8]. Importantly, EF is associated with the development of the frontal area of the brain, an area that continues to mature and develop throughout adolescence and into early adulthood [9,10]. Thus, any disturbance to the development of the frontal region during this critical period (for example because of an episode of depression) can have a long-lasting impact, and may explain why depression that occurs during adolescence and early adulthood is associated with long-term impairments into adult life [11,12].

Flavonoids are a class of polyphenols (micronutrients) found naturally in fruits, vegetables, tea, coffee and cocoa. Flavonoid consumption has been associated with both vascular and cognitive benefits across the lifespan [13,14,15,16,17]. Single-dose flavonoid interventions have produced improvements in attention, inhibition, visuospatial memory, and executive function between 2–6 h post-consumption [14,18,19,20], whilst supplementation of flavonoids for 1.5–8 weeks has been associated with improved visuospatial memory and improved long-term memory [15,21,22]. Numerous mechanisms of action have been investigated to explain the beneficial effects of flavonoids on cognition. These include increases in cerebral blood flow, protecting against neuronal stress via anti-inflammatory and anti-oxidative effects, and positively stimulating neural signaling pathways, such as Extracellular Signal-Regulated Kinase (ERK), Serine/Threonine-specific Protein Kinase (Akt) and Brain-Derived Neurotrophic Factor (BDNF), leading to improved neural signaling [15,21,23,24].

Independently of the experimental evidence that shows flavonoids improve cognitive performance, there is emerging evidence that flavonoids may also support mental health and well-being. Epidemiological data shows that lifetime consumption of fruit and vegetables (and therefore higher flavonoid consumption) predicts a lower incidence of depression in later life [25,26,27,28,29]. The benefits are also seen earlier in life; a recent systematic review concluded that whilst the quality of evidence was weak, there was a consistent body of research reporting cross-sectional and longitudinal associations between nutrition and mental health in children and young people [30]. Similar findings have been shown by other authors [31,32]. However, there is an absence of studies exploring the effects of flavonoid-rich interventions on mood.

Given the well-documented links between flavonoid consumption and cognition, and between cognition and depression, the studies reported in this paper assess the acute effects of flavonoid-rich wild blueberries (WBB) on mood two hours post-consumption. This two-hour interval coincides with the time-frame for the peak absorption and metabolism of the anthocyanins present in blueberries [33]. In addition, it is important to establish whether acute effects on mood are observable prior to considering a chronic flavonoid-based intervention for mood outcomes. Two independent groups, healthy children and young adults, were recruited. These groups represent individuals who are at crucial stages of mental and cognitive development and thus plausible points at which prevention and public health interventions may be particularly powerful.

## 2. Materials and Methods

The research was reviewed and given a favorable ethical opinion for conduct by the University of Reading Research Ethics Committee (2015-148-CW & UREC 15/10) and was conducted in accordance with the Declaration of Helsinki. All participants were screened for food related allergies or other health conditions, e.g., diabetes, heart disease, blood pressure, thyroid, kidney and liver diseases which would exclude them from the study.

### 2.1. Study 1 (Young Adults)

Participants: 21 undergraduate students were recruited from University of Reading; 19 females and two males aged between 18 and 21 years (Mean (M) = 20.14 years, Standard Deviation (SD) = 1.01).

Drink preparation and consumption: All interventions were prepared on site, no more than 20 min before consumption, by an independent researcher who did not administer the drink to participants. The flavonoid-rich wild blueberry (WBB) drink contained 253 mg anthocyanins and was prepared by mixing 30 g of freeze-dried WBB with 30 mL of low-flavonoid Rocks Orange Squash and 220 mL of water. The placebo drink was matched to the WBB drink for vitamin C (4 mg), sugars (8.90 g fructose, 7.99 g glucose), 30 mL Rocks Orange Squash and 220 mL of water. Drinks were prepared in an opaque cup and straw to ensure that double-blinding was maintained.

Mood Measure: The Positive and Negative Affect Schedule-NOW (PANAS-NOW) was used to assess current mood. The PANAS-NOW is a valid and reliable 20-item (10 positive and 10 negative mood states) self-report measure of Positive Affect (PA) and Negative Affect (NA) [34,35] which can be used on multiple test occasions. Participants were asked to rate the degree to which they were currently experiencing each item, on a five-point Likert scale. The ratings of positive and negative items were summed to calculate an overall positive and overall negative affect score, ranging from 10–50 (lower scores indicating lower levels of positive or negative affect). A cognitive performance assessment (Modified Flanker Task; MFT) was also administered after each presentation of the PANAS, the data for which will be reported elsewhere.

Procedure: This was a double-blind, placebo-controlled, crossover study. Informed consent was obtained from all participants for inclusion before they participated in the study. To minimise variability caused by prior flavonoid consumption, participants were given a list of high polyphenol food items (such as tea, coffee, chocolates, most fruit and vegetables) and asked not to consume these for 24 h before each test session, including the morning of the sessions. All test sessions were scheduled in the morning and all participants attended three test sessions separated by a minimum three-day wash-out period (range three to seven days; median three days). This included a practice test day (Screening), which was described to the participants as the first test day, where all participants received the placebo drink. This was to ensure participants were fully versed in the experimental procedures before testing began. Thereafter they were tested on two further occasions, during which the placebo or flavonoid intervention was administered to each individual in a random order (10 received WBB first).

On each of the three visits to the laboratory, participants completed the baseline mood measure (PANAS-NOW) on arrival. Immediately following this they consumed the drink. They were asked to return to the laboratory 2 h later and refrain from eating, exercising or drinking during this time. However, water consumption was permitted before and during the 2 h break. Upon their return, participants completed the PANAS-NOW again. Overall, each visit to the laboratory lasted approximately 30 min.

### 2.2. Study 2 (Children)

Participants: 52 participants (29 female) aged seven to ten years old (Mean age = 8.241, SD = 0.965) were recruited from two local primary schools in Berkshire, UK.

Drink preparation and consumption: On the test day, participants were randomly allocated to receive the wild blueberry drink (*n* = 28; 17 females; Mean age = 8.236, SD = 0.869) or a matched placebo (*n* = 24; 12 females; Mean age = 8.227, SD = 1.031). The contents of each drink were identical to Study 1, but instead only included 170 mL of water to aid consumption and palatability in a child population. A confederate prepared all drinks at the school immediately before they were administered to the participants by the researcher. Drinks were offered in an opaque drinking cup with a black straw placed through the lid to ensure double blinding.

Measures: A computerised version of the children’s Raven’s Coloured Progressive Matrices (RCPM) was administered at screening to measure fluid intelligence. This is a well validated measure assessing non-verbal and reasoning abilities [36]. This measure was included to ensure all participants were of healthy cognitive functioning for their age, and to ascertain intervention effects in relation to intelligence. The York Assessment of Reading Comprehension was administered to ascertain whether participants were at an age-appropriate reading comprehension level. A modified Continuous Performance Task was also employed to assess possible attentional deficits. Data from these three tasks were not used in the analysis of the current study. A practice of the child version of the Positive and Negative Affect Scale (PANAS-C) and cognitive task battery was also undertaken to ensure understanding of the tasks and reduce practice effects. This practice data was not analysed.

For the main test sessions, the PANAS-C was administered alongside a 40 min cognitive battery which included Rey’s Auditory Verbal Learning Task (RAVLT), Modified Flanker Task (MFT) and Test of Word Reading Efficiency (TOWRE-2) [37,38,39]. The cognitive performance data is reported elsewhere [40].

PA and NA were calculated by summing positive and negative items using the validated children’s version of the PANAS; PANAS-C [36]. The PANAS-C has 30 items (15 positive and 15 negative emotions) and includes the original 20 items from PANAS-NOW and 10 additional child-friendly synonym items derived from the PANAS-X (Expanded Form). This was administered and analysed as in study one.

Procedure: This study was also double-blind and placebo-controlled. A between-groups design was used to minimise disruption to the school and demand on participants. All children took part in a screening session one to two days before the main test day. The main test day consisted of a baseline session and a post-consumption session 2 h later. All parents or legal guardians gave written consent for their child to take part, and each child gave verbal assent before any research began. Parents confirmed that their child had no allergies or food intolerances that would prevent them from taking part. To accommodate school hours, children were tested during the afternoon at school. They were not required to fast prior to testing. However, parents were asked to make sure that their child consumed a low-flavonoid diet for 24 h before the baseline session, including breakfast and lunch on the main test day. Parents were telephoned after their child’s screening session to give them the date of their child’s next test day, and to remind them about the dietary restrictions. School canteen staff members were also asked to monitor children’s meals on the day of testing and to remind the children taking part in the study not to eat high-flavonoid foods for lunch that day.

Children were tested individually in a quiet space at school on two separate occasions, one to two days apart. On day one, children completed the screening and practice tasks outlined previously. On testing day 2, after a low-flavonoid lunch, participants completed the PANAS-C and cognitive battery (data reported elsewhere [40]), before consuming the placebo or flavonoid drink. They then returned to their classrooms and were asked not to exercise or consume anything except water. After 2 h, participants individually completed the PANAS-C and a matched version of the cognitive battery again in the presence of the researcher. Participants were given a written debrief about the study aims and were given similar debrief information to take home to their parents.

### 2.3. Analysis

Data was analysed using SPSS (Version 22.0). In both studies, PA and NA were dependent variables in a two-way analysis of variance (ANOVA) with Drink (placebo, WBB) and Session (pre- and post-consumption) as independent variables. For study 1, this was a fully repeated measures 2 × 2 ANOVA (as participant consumed both drinks and were tested at all time points), and for study 2 this was a mixed 2 × 2 ANOVA (as participants consumed either the placebo or WBB, and were tested at both time points). Significant main effects and interactions were explored with Bonferroni corrected post-hoc t-tests. Baseline differences in intelligence quotient (IQ (study 2) were examined using one-way ANOVAs with Drink (placebo and WBB) as the independent variable.

## 3. Results

### 3.1. Study 1 (Young Adults)

**Figure 1:**
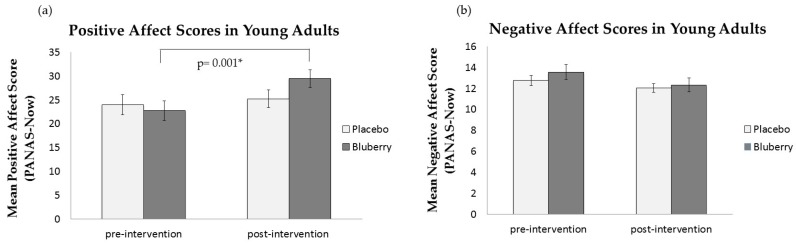
Mean PANAS-NOW Mood scores in adults aged 18–21 years: (**a**) Mean PA scores pre- and post-consumption of placebo and WBB drinks; (**b**) Mean NA scores pre- and post-consumption of placebo and WBB drinks.

Figure 1 shows PA and NA before and after consumption of the placebo and WBB drinks. There was no significant main effect of Drink on positive affect (*F*(1,20) = 1.24, *p* = 0.28). There was a significant main effect of Session (*F*(1,20) = 10.67, *p* = 0.004) and a significant Drink × Session interaction (*F*(1,20) = 7.5, *p* = 0.013). Subsequent Bonferroni-corrected post-hoc t-tests demonstrated that there was a significant increase in PA after consuming the WBB drink (pre: M = 22.76, SD = 9.01 and post: M = 29.48, SD = 8.60; *t*(20) = −4.68, *p* < 0.001). There was no change in PA after consuming the placebo drink (pre: M = 24.0, SD = 9.59 and post: M = 25.24, SD = 8.50; *t*(20) = −0.73, *p* = 0.48, Figure 1a). Post-hoc tests showed no significance difference in PA and NA between drinks at baseline (*p* > 0.05) but there was a significant difference in PA scores post-consumption between the placebo and WBB drink (*t*(20) = 2.286, *p* = 0.033). The main effect of Session was explained by an increase in PA post-consumption (M = 27.36, SD = 8.55) relative to pre-consumption (M = 23.38, SD = 9.3), which, as indicated by the significant interaction, was driven by the WBB drink.

There was a significant main effect of Session on NA (*F*(1,20) = 8.30, *p* = 0.009). After consuming both placebo and WBB drinks, the participants reported a reduction in NA (pre: M = 13.17, SD = 2.67 and post: M = 12.19, SD = 2.49; Figure 1b). This may be explained by postprandial blood glucose effects due to the matched sugar content of both drinks. There was no significant main effect of Drink on NA (*F*(1,20) = 0.67, *p* = 0.42) and no significant Drink x Session interaction on NA (*F*(1,20) = 0.51, *p* = 0.49).

### 3.2. Study 2 (Children)

Forty-nine participants were included in the analysis of IQ due to three missing data files. There were no significant differences at baseline between groups for IQ (Ravens: WBB group, M = 26.78, SD = 4.31; Placebo group, M = 26.55, SD = 5. 98; *F*(1,47) = 0.024, *p* = 0.878), PA (WBB group, M = 49.00, SD = 11.96; Placebo group, M = 49.21, SD = 10.04; *F*(1,50) = 0.005, *p* = 0.947) or NA (WBB group, M = 20.79, SD = 7.43; Placebo group, M = 19.25, SD = 3.86; *F*(1,50) = 0.832, *p* = 0.366).

**Figure 2:**
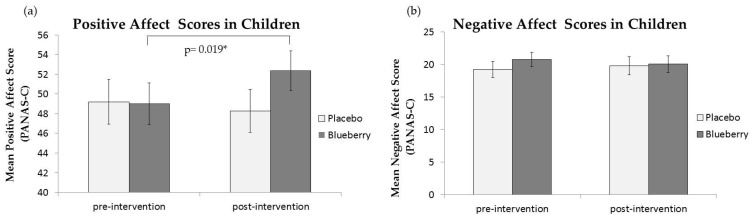
Mean PANAS-C scores in children aged 7–10 years: (**a**) Mean PA scores pre- and post-consumption of placebo and WBB drinks; (**b**) Mean NA scores pre- and post-consumption of placebo and WBB drinks. * Significant at <0.05. Attained from post-hoc paired samples *t*-test.

Figure 2 shows PA and NA in both groups before and after the intervention. There was no significant main effect of Session (*F*(1,50) = 1.362, *p* = 0.249) or Drink (*F*(1,50) = 0.456, *p* = 0.503) for PA. However, there was a significant Session × Drink interaction (*F*(1,50) = 4.176, *p* = 0.046). Paired samples *t*-tests for each condition revealed no significant change in PA after consuming the placebo drink (pre: M = 49.21, SD = 16.04 and post: M = 48.29, SD = 15.51; *t*(23) = 0.564, *p* = 0.578), but a significant increase in PA after consuming the WBB drink (pre: M = 49.0, SD = 14.86 and post: M = 52.36, SD = 14.36; *t*(27) = −2.495, *p* = 0.019; Figure 2a). There was no significant difference in PA between placebo and WBB at the post-consumption time point (*t*(52) = −1.597, *p* = 0.116).

Negative Affect in both groups is shown in Figure 2b. There was no significant main effect of Session (*F*(1,50) = 0.009, *p* = 0.927) or Drink (*F*(1,50) = 0.355, *p* = 0.554) on NA. There was also no significant Session × Drink interaction (*F*(1,50) = 0.453, *p* = 0.504; Figure 2b). NA did not change 2 h after consuming either the placebo (pre: M = 19.25, SD = 8.73 and post: M = 19.79, SD = 9.78) or the WBB drink (pre: M = 20.79, SD = 8.09 and post: M = 20.07, SD = 9.06).

## 4. Discussion

These randomised, placebo-controlled, double-blind studies investigated the effects of acute consumption of a flavonoid-rich wild blueberry drink on the mood of healthy children and young adults. In both studies, increased Positive Affect was observed 2 h after consumption of the flavonoid-rich WBB drink (significant drink by session interaction). The flavonoid drink had no effect on Negative Affect. The effect of flavonoids on mood was consistent across two populations, at two different time points (morning and afternoon), and in a between- and within-subject design. Thus, the positive effect of blueberry flavonoids on Positive Affect appears to be robust to variations in experimental design.

The distinctive effect of flavonoids on PA but not NA is notable. PA and NA reflect orthogonal facets of mood. A low PA is more highly linked to depression, and high NA is more closely related to anxiety [34,35,36]. Thus, these data suggest that the effect of flavonoid consumption on mood may be specific to depressive disorders, rather than pervasive across different mood states. In the young adult study, both drinks led to a decrease in NA. This may be due to the sugar content of both the drinks, as dietary carbohydrates have been shown to enhance the uptake of circulating tryptophan (a precursor of serotonin) into the brain by promoting insulin secretion [41,42].

Although preliminary, these results are intriguing and warrant focused investigation of the relationship between flavonoids and mood, as well as with mental health more generally. It is important to note that diagnosis of mental health disorders or consumption of medication were not specific exclusion criteria; however, the data showed normal levels of PA and NA [34,43], indicating a healthy sample. The young adult participants were predominantly female, and therefore these results may not be generalisable to a male population; however, there is no evidence to suggest a gender-specific mechanism underlying the effects of flavonoids on the brain. The child sample was of average IQ, and the young adult population was recruited from a University population and thus likely to have an average to above-average IQ. No carry-over effects of flavonoids are expected as the half-life of flavonoids is estimated to range from 2–28 h and there was a minimum of three days insured between the test days [44].

Mood is by definition a short-term experience. In non-clinical populations mood is usually labile. However, sustained periods of low mood (dysphoria) are a strong predictor of the emergence of major depressive disorder. Therefore, if acute flavonoid consumption improves Positive Affect, sustained consumption of flavonoids may help prevent dysphoria, and thus, major depression. Given that depression tends to emerge for the first time during adolescence or early adulthood, and is likely to reemerge as a relapse later in life, an intervention which increases flavonoid consumption during this critical period of development could decrease the incidence of adolescent and life-long depression.

The mechanism linking flavonoids and mood is not known and requires greater consideration. There are a number of plausible mechanisms which may explain these results. First is the finding that flavonoids increase cerebral blood flow [45]. One of the last brain structures to mature is the dorsolateral prefrontal cortex (DLPFC), a site within the frontal lobes highly associated with cognitive control [46] and emotional regulation [47]. Increased cerebral blood flow to this area may help strengthen neural circuitry in the frontal lobes, where cognitive and emotional control is located. This is consistent with the evidence linking executive functioning with low mood, and suggests an indirect pathway whereby flavonoid consumption enhances cerebral blood flow, boosting executive functioning, and thus helping to inhibit cognitive features (i.e., rumination) that maintain depression.

A second, alternative explanation for the link between flavonoids and mood is the effect of anthocyanins (a subtype of flavonoids) on Monoamine Oxidase (MAO) inhibition. MAO is involved in the oxidation of monoamines, some of which are neurotransmitters involved in the regulation of mood (e.g., serotonin, dopamine, and noradrenaline). MAO inhibitors have been used to treat mood disorders. Thus, the consumption of fruits high in flavonoids, such as blackcurrants, may significantly reduce MAO activity, thereby increasing circulating monoamines, and elevating mood [48]. This would suggest a direct pathway between flavonoid consumption and mood.

A third possible mechanism is the ability for flavonoids to mimic anxiolytic-like effects by binding to benzodiazepine receptors, which influences the effects of Gamma Amino Butyric Acid (GABA) via GABA_A_ receptors [49,50]. GABA is an inhibitory neurotransmitter present exclusively in the central nervous system. In addition to regulating cognition, decreased levels of GABA are associated with mood disorders. However, in the two studies reported here, the absence of an effect of flavonoid consumption on NA (a strong indicator of anxiety) suggests that the GABA hypothesis may not be a plausible explanation for the observed acute effects on PA.

## 5. Conclusions

This study demonstrated acute effects of blueberry flavonoid consumption on Positive Affect and no effect on Negative Affect in healthy children and young adults. Dietary interventions could play a key role in promoting positive mood and are a possible way to prevent dysphoria and depression. Given the potential implications of these findings for preventing depression, a disabling and common mental health problem in adolescents and adults, it is important to replicate the study and assess the potential to translate these findings to practical, cost-effective and acceptable interventions.
